# Snacking patterns, diet quality, and cardiovascular risk factors in adults

**DOI:** 10.1186/1471-2458-14-388

**Published:** 2014-04-23

**Authors:** Theresa A Nicklas, Carol E O’Neil, Victor L Fulgoni III

**Affiliations:** 1 Department of Pediatrics, Baylor College of Medicine, USDA/ARS Children’s Nutrition Research Center, 1100 Bates Ave, Houston, TX 77030, USA; 2 School of Nutrition and Food Sciences, Louisiana State University Agricultural Center, 261 Knapp Hall, 110 LSU Union Square, Baton Rouge, LA 70803, USA; 3 Nutrition Impact LLC, 9725 D Drive North, Battle Creek, MI 49014, USA

**Keywords:** Adults, Snacking patterns, Overweight, Abdominal obesity, Cardiovascular risk factors, Diet quality

## Abstract

**Background:**

The relationship of snacking patterns on nutrient intake and cardiovascular risk factors (CVRF) in adults is unknown. The aim of this study was to examine the associations of snacking patterns with nutrient intake, diet quality, and a selection of CVRF in adults participating in the 2001-2008 National Health and Nutrition Examination Survey.

**Methods:**

24-hour dietary recalls were used to determine intake and cluster analysis was used to identify the snacking patterns. Height and weight were obtained and the health indices that were evaluated included diastolic and systolic blood pressure, high density lipoprotein-cholesterol, low density lipoprotein cholesterol, triacylglycerides, blood glucose, and insulin.

**Results:**

The sample was participants (n = 18,988) 19+ years (50% males; 11% African-Americans; 72% white, 12% Hispanic-Americans, and 5% other). Cluster analyses generated 12 distinct snacking patterns, explaining 61% of the variance in snacking. Comparisons of snacking patterns were made to the no snack pattern. It was found that miscellaneous snacks constituted the most common snacking pattern (17%) followed by cakes/cookies/pastries (12%) and sweets (9%). Most snacking patterns were associated with higher energy intakes. Snacking patterns cakes/cookies/pastries, vegetables/legumes, crackers/salty snacks, other grains and whole fruit were associated with lower intakes of saturated fatty acids. Added sugars intakes were higher in the cakes/cookies/pastries, sweets, milk desserts, and soft drinks patterns. Five snack patterns (cakes/cookies/pastries, sweets, vegetable/legumes, milk desserts, soft drinks) were associated with lower sodium intakes. Several snack patterns were associated with higher intakes of potassium, calcium, fiber, vitamin A, and magnesium. Five snacking patterns (miscellaneous snacks; vegetables/legumes; crackers/salty snacks; other grains; and whole fruit) were associated with better diet quality scores. Alcohol was associated with a lower body mass index and milk desserts were associated with a lower waist circumference. No snack patterns were associated with other CVRF studied.

**Conclusions:**

Overall, several snacking patterns were associated with better diet quality than those consuming no snacks. Yet, the majority of the snacking patterns were not associated with CVRF. Education is needed to improve snacking patterns in terms of nutrients to limit in the diet along with more nutrient-dense foods to be included in snacks.

## Background

In 2009-2010, 36% of adults in the US were obese [1]. Obesity increases the risk of a number of health conditions including hypertension, dyslipidemia, type 2 diabetes, and metabolic syndrome [2,3]. Obesity also affects quality of life, increases medical cost, and increases job absenteeism in adults [4-8]. The direct and indirect cost associated with obesity in adults is estimated at $209 billion or 21% of US healthcare expenditures [9].

Snacking has become ubiquitous in the American society. The percentage of adults consuming snacks increased from 71% in 1977-1978 to 97% in 2003-2006 [10]. The number of snacking occasions increased 0.97 events over this same time period and the contribution of snacks to total energy intake increased from 18% to 24% [10]. Snacking has been shown to be associated with increased energy intake [11]. The increased energy intake associated with snacking may reflect the energy density [12] and portion sizes of many foods and beverages consumed as snacks [13-15]. Snacking also contributed significantly to nutrient intake [11,16,17], better diet quality [18], and increased likelihood of meeting selected national recommendations [11,16].

Studies have suggested that several characteristics of dietary behavior such as eating frequency or snacking [19-21] may influence body weight. The Booth Hypothesis [22] stated that “grazing” or multiple eating episodes between meals, rather than the traditional pattern of three meals per day, was a major factor that contributed to obesity. Contrary to this hypothesis, few adult studies have shown that snacking was positively [23] or negatively associated with body fatness [19,24] or reduced risk of overweight and abdominal obesity [25,26]. Others have shown that snacking was not associated with weight [11,24,26-28] and was not an independent predictor of weight gain [29]. Results may be equivocal because snack definitions have not been clearly established, thus were not consistent across studies [12,30-32]. The snacking studies were also based on the assumption that snacking patterns were not unique in their contribution to nutrient intake. There are several possible explanations for the lack of association between snacking patterns and weight, despite the increased energy intake associated with the snacking patterns. Snacking has been shown to be associated with improved diet quality [11,14,33] and increased intakes of fruit, whole grains, and fiber [11,14], which could promote satiety and reduce risks for obesity. Snacking has also been associated with increased vigorous physical activity [16,34]; thus, the increased energy intake associated with snacking may have been compensated for by increased energy expenditure during physical activity. A lack of association between snacking and weight could also be explained if overweight individuals who try to lose weight avoid eating snacks. More studies are needed to better understand the mechanisms by which snacking may impact the balance of energy intake and energy expenditure.

Few studies have attempted to examine the association of snacking with specific cardiometabolic risk factors [35-39]. Majority of these studies were limited to foreign populations of male and/or female adults or adolescents and snacks were not uniformly defined across these studies and were typically included as a component of food patterns or dietary recommendations. Snack foods were determined using a food frequency questionnaire with a limited number of snack foods assessed. None of these studies looked at snacking patterns, which can vary considerably, with cardiovascular risk factors (CVRF) in a nationally representative population of US adults. To our knowledge this is the first study to examine the various snacking patterns among adults and their impact on nutrient intake, diet quality, and a selection of CVRF (including overweight/obesity).

## Methods

### Study overview, population, and analytic sample

Data from adults 19+ years of age (y) (n = 18,988) participating in the National Health and Nutrition Examination Survey (NHANES) 2001-2008 were combined for these analyses to increase the sample size [40]. This was a secondary data analysis with a lack of personal identifiers; therefore, this study was exempted by the Baylor College of Medicine Institutional Review Board.

### Dietary and physical activity assessment

Dietary intake data were obtained from in-person 24-hour dietary recall interviews (Day 1) using an Automated Multiple-Pass Method [41] in the Mobile Examination Center (MEC). The Multiple-Pass Method [42-44] consisted of five steps: (a) the quick list, which was an uninterrupted listing by the subject of foods and beverages consumed; (b) the forgotten foods list, which queried the subject on categories of foods that have been documented as frequently forgotten; (c) a time and occasion at which foods were consumed; (d) the detail cycle, which elicited descriptions of foods and amounts eaten aided by the interactive use of a Food Model Booklet and measuring guides; and finally, (e) the final probe review. For data collection years 2001-2002, only a single 24-hour dietary recall was collected. Although two 24-hour dietary recalls were collected in 2003-2008, only data from the first recall was used to assess snacking patterns. There was a concern that differences in methodology might confound the results. Adults whose 24-hour recall data were judged to be incomplete or unreliable by staff of the National Center for Health Statistics (Hyattsville, MD) were excluded from these analyses. Females who were pregnant or lactating were also excluded. Snacks were self-defined by subjects as eating occasions with foods or beverages not consumed with meals. The timing of eating meals was defined in the 24-hour recall. Detailed descriptions of the dietary recalls and data collection are available in the NHANES Dietary Interviewer’s Training Manual [45].

Energy and nutrient intakes were calculated using the USDA’s Food and Nutrient Database for Dietary Studies (versions 1.0 – 4.1) [46], for NHANES 2001-2002, 2003-2004, 2005-2006, and 2007-2008. The nutrients studied reflect the nutrients to limit in the diet (i.e. total energy, saturated fatty acids, added sugars and sodium), nutrients of public health concern (i.e. potassium, calcium, vitamin D, and fiber), and nutrients under-consumed (i.e. vitamin A, vitamin C, vitamin K, folate, and magnesium), as defined by the 2010 Dietary Guidelines for Americans [47]. The MyPyramid Equivalents Database (MPED), versions 1.0 [48] and 2.0 [49], was used to examine consumption in terms of MyPyramid [49] food group equivalents. The MPED translates dietary recall data into equivalent servings of the seven MyPyramid major food groups and corresponding subgroups. The number of MyPyramid food group equivalent servings was based on the 24-hr food dietary recall data from NHANES 2001-2008. Foods were hand matched to the same/similar foods for NHANES 2003-2008 since these data were released without an update to the MPED.

Diet quality was calculated using the Healthy Eating Index-2005 (HEI-2005) [50,51]. Food group standards and the development and evaluation of the HEI-2005 have been previously described [52,53]. The SAS code used to calculate HEI-2005 scores was downloaded from the Center for Nutrition Policy and Promotion website [54]. Briefly, HEI-2005 was designed to evaluate all of the major MyPyramid food groups and major subgroups. The 12 HEI-2005 components were summed for a total possible score of 100 points. Each participant’s component score was calculated by dividing the total component intake by the total energy intake and multiplying by 1000. Scores were energy-adjusted on a density basis (per 4187 kJ), which allowed for characterization of diet quality while controlling for diet quantity. Physical activity was determined using a questionnaire [55] that assessed sedentary, moderate and vigorous physical activity in a typical week.

### Physiologic measures

Height and weight were obtained according to NHANES Anthropometry Procedures Manual [56]. The manual provides information about equipment, calibration, methods, quality control, and survey procedures. Anthropometry data was measured data by study researchers in NHANES. Body mass index was calculated as body weight (in kilograms) divided by height (in meters) squared [57]. Waist circumference (WC) was obtained using NHANES protocols [56].

### Cardiovascular risk factors

Health indices that were evaluated included diastolic (DBP) and systolic blood pressure (SBP), high density lipoprotein-cholesterol (HDL-C), low density lipoprotein cholesterol (LDL-C), triacylglycerides (TAG), blood glucose, and insulin. Measurements of CVRF were obtained in the MEC according to the NHANES protocols [56,58]. Three or four readings for SBP and DBP were recorded in the NHANES; an average from each set of readings was used in this study. Venous blood was drawn in the MEC and total HDL-C were determined on non-fasted individuals (n = 18,988) and LDL-C, TAG, and blood glucose were determined on only fasted subjects (n = 8,099); thus, not all individuals had laboratory values for all tests. Plasma glucose was measured spectrophotometrically using a series of enzymatic reactions (Roche Diagnostics, IN) [59]. Serum LDL-C was calculated according to the Friedewald equation and was reported only for fasting participants [59]. Serum HDL-C was measured using enzymatic reactions in conjunction with the heparin-manganese precipitation method or a direct immunoassay technique (Roche Diagnostics, IN) [59].

### Clinical definitions

Overweight/obesity was defined as a BMI ≥ 25 [60]; elevated waist circumference, WC ≥ 102 cm (males) or ≥88 cm (females); elevated blood pressure, SBP ≥ 130 mmHg or DBP ≥85 mmHg or antihypertensive medication use; reduced serum HDL-C, <2.22 mmol/l (males) or <2.77 mmol/l (females) or medication use for reduced HDL-C; elevated serum LDL-C, ≥ 3.37 mmol/l; elevated serum TAG, ≥ 8.33 mmol/l or medication use for elevated TAG; elevated fasting plasma or serum glucose, ≥5.55 mmol/l or medication use for elevated glucose. Data on medication use were obtained from the NHANES household interview on prescription medications or from questionnaires pertaining to blood pressure and diabetes mellitus. Abnormal values for other cardiovascular risk factors (CVRF) were determined using established criteria [61-65].

### Cluster analysis

Snacking intake patterns were identified using SAS 9.2 (SAS Institute, Cary, NC, 2009) PROC CLUSTER using a single 24-hour dietary recall in NHANES 2001-2008. NHANES population weights were applied. Cluster analyses allow the user to focus on a particular defined aspect (e.g. snacking calories) and then forces maximal differences in clusters for assessment. Cluster analysis also allows for group comparisons rather than factor analysis which are generally associations. For these analyses, the USDA food groups were collapsed into 21 snacking food groupings. All food codes fit in one and only one of the snacking food groupings. The patterns identified by the cluster analysis were then identified by percent calories within each snacking food grouping (only foods that contributed 5% or more of calories were included) at the centroid of each cluster. Using this method resulted in 12 readily identifiable snacking patterns, such as crackers/salty snacks, sweets, fruit; no snacks was one of the 12 patterns identified. With snacking patterns identified, each participant was placed into one snacking pattern. A subject was placed in one of 12 snacking patterns based on the percent of calories from snacks for the day falling in each of the distinct food categories. The cluster definitions and the associations of subjects with a cluster are directly from the output from the cluster procedure and each subject was then placed in the cluster that matched most closely to the pattern of calories across the food categories.

### Statistical analyses

For the initial analyses, SUDAAN v10.0 (Research Triangle Institute; Raleigh, NC) was used to adjust analyses for sampling weights and the sampling units and strata information as provided by NHANES. Dietary day 1 weights were used for all analyses. Least-square means ± SE were calculated using PROC REGRESS of SUDAAN for dietary intake, diet quality (HEI-2005), and physiological measures were determined for participants consuming each snacking pattern. Covariates included age, gender, and race/ethnicity. The poverty income ratio (PIR) grouped into three categories (<1.25, 1.25–3.49, and >3.49), physical activity level (sedentary, moderate and vigorous), alcohol intake (g/d), and energy intake for nutrient related variables (not for energy intake itself, HEI-2005, or physiological measures) also served as covariates. The PIR values reflected the federally set poverty lines, so a PIR of <1.25 equated to below 125% of poverty. Higher values mean the individuals had higher incomes. The HEI-2005 was not controlled for energy intake, since it is already controlled for energy [66]. Statistical differences for variables of interest were determined via *t*-test comparing to the no snacking group.

Odds ratios and Bonferroni corrected 95^th^ percentile upper and lower confidence intervals for being overweight, being obese, and having other cardiovascular risk factors were evaluated with no snacks as the reference group using the PROC LOGISTIC procedure of SUDAAN software controlling for the above mentioned covariates. A probability of p ≤ 0.05 was considered significant; however, a Bonferroni correction was applied for multiple comparisons (0.05/12), so the effective p value was p ≤ 0.0042.

## Results

### Demographics of the snacking patterns

The demographic characteristics of the sample for each of the 12 snacking patterns are presented in Table 1. The total sample (n = 18,988) was aged 19 y and older (50% males; 11% African-Americans; 72% whites; 12% Hispanic-American and 5% other) with a mean poverty ratio of 3.02; 37% of the population reported doing moderate-vigorous physical activity. Thirteen percent reported consuming no snacks.

**Table 1:** Demographics of the sample by snacking patterns in adults ≥19 years of age

			**Snacking patterns**											
**Demographics %**		**Total sample (n = 18,988)**	**Misc. snacks (n = 3,230)**	No snacks** (n = 2,853)	**Cakes/Cookies/Pastries (n = 2,180)**	**Sweets (n = 1,495)**	**Vegetables/Legumes (n = 1,524)**	**Alcohol (n = 1,572)**	**Milk desserts (n = 1,355)**	**Crackers/Salty snacks (n = 1,293)**	**Soft drinks (n = 1,088)**	**Other grains (n = 1,190)**	**Whole fruit (n = 913)**	**Coffee/Tea (n = 295)**
Total		100	17	13	12	9	8	8	8	7	6	6	4	2
Gender														
Male		49	48	42	45	41	48	67	48	44	56	50	44	48
Female		51	52	48	55	59	52	33	52	56	44	50	56	52
Ethnicity														
White		72	67	64	78	75	76	74	82	77	74	64	63	75
African-American		11	14	13	11	10	10	13	8	11	10	11	10	6
Hispanic-American		12	13	17	8	10	9	9	7	9	11	19	20	10
Other		5	6	6	4	5	5	4	4	3	5	5	7	9
Physical Act.														
Sedentary		31	31	36	31	30	28	26	31	28	34	29	31	29
Light		33	31	33	35	31	36	31	33	33	29	33	34	32
Mod-Vig		37	37	32	34	39	36	44	36	39	37	38	35	39
PIR*		3	3	3	3	3	3	3	3	3	3	3	3	3

*PIR = Poverty Income Ratio.

**Reference group in No Snacks.

### Description of snacking patterns

**Figure 1:**
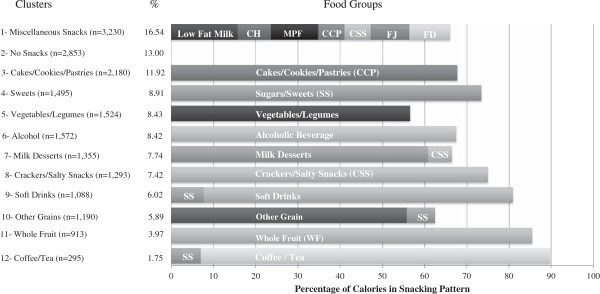
**Description of the twelve snacking pattern clusters in adults ≥19 years of age.** Reference group is Cluster Pattern #2 - No Snacks (n = 2853, 13%). SS = Sugars and Sweets; WF = Whole Fruit; CSS = Crackers/Salty Snacks; CH = Cheese; MPF = Meat/Poultry/Fish; CCP = Cakes/Cookies/Pastries; FJ = Fruit Juice; FD = Fruit Drinks.

The 12 snacking patterns (n and% of population) were: miscellaneous snacks (n = 3,230, 17%), which included fruit juice, fruit drinks, meat/poultry/fish, cheese, low-fat milk, cakes/cookies/pastries, and crackers/salty snacks; no snacks (n = 2,853, 13%); cakes/cookies/pastries (n = 2,180, 12%); sweets (n = 1,495, 9%); vegetables/legumes (n = 1,524, 8%); alcohol (n = 1,572, 8%); milk desserts (n = 1,355, 8%); crackers/salty snacks (n = 1,293, 7%); soft drinks (n = 1,088, 6%); other grains (n = 1,190, 6%); whole fruit (n = 913, 4%); and, coffee/tea (n = 295, 2%). A description of the foods in the snacking pattern clusters is provided in Figure 1.

### Energy and nutrients to limit

Table 2 shows mean intakes of energy and nutrients to limit, including saturated fatty acid (SFA), solid fat, added sugars, and sodium by snacking patterns for the entire day. Most of the snacking patterns were associated with a higher total energy intake, with the exception of the alcohol, whole fruit, and coffee/tea snacking patterns when compared to no snacks. Total energy intake of the snacking patterns ranged from 7,939 kJ (no snacks) to 10,153 kJ (other grains).

**Table 2:** **Consumption of energy and nutrients to limit by snacking patterns**^**1 **^**in adults ≥19 years of age**

	**Snacking Patterns LSM (SE)**											
**Energy & nutrients to limit**	**Misc. snacks (n = 3,230)**	No snacks** (n = 2,853)	**Cakes/Cookies/Pastries (n = 2,180)**	**Sweets (n = 1,495)**	**Vegetables/Legumes (n = 1,524)**	**Alcohol (n = 1,572)**	**Milk desserts (n = 1,355)**	**Crackers/Salty snacks (n = 1,293)**	**Soft drinks (n = 1,088)**	**Other grains (n = 1,190)**	**Whole fruit (n = 913)**	**Coffee/Tea (n = 295)**
Total energy (kJ)^1^	9538.66 (100.07)*	7936.84 (107.69)	9959.07 (104.13)*	9301.59 (135.45)*	9812.11 (134.32)*	8206.73 (145.58)	9767.43 (145.75)*	8968.55 (130.09)*	8908.05 (179.45)	10152.81 (205.96)*	8083.38 (146.63)	8455.73 (287.48)
Saturated fatty acid (g)^1^	27.59 (0.27)	28.03 (0.27)	26.61 (0.30)*	28.55 (0.32)	26.37 (0.32)*	27.60 (0.39)	30.70 (0.39)*	26.02 (0.29)*	25.74 (0.42)*	25.80 (0.45)*	25.98 (0.21)*	28.37 (0.99)
Solid fat (g)^1^	46.91 (0.52)	49.05 (0.65)	49.83 (0.57)	45.83 (0.61)*	39.49 (0.74)*	47.82 (0.78)	50.64 (0.67)	45.11 (0.75)*	44.97 (0.81)*	45.22 (0.96)*	44.95 (0.89)*	47.87 (1.43)
Added sugars (tsp.)^1^	17.94 (0.40)	18.68 (0.51)	23.17 (0.52)*	22.77 (0.50)*	15.61 (0.58)*	18.93 (0.59)	21.58 (0.55)*	16.66 (0.51)*	28.74 (0.73)*	16.35 (0.56)*	15.13 (0.46)*	18.63 (1.08)
Sodium (mg)^1^	3568.88 (27.24)	3626.67 (36.25)	3321.33 (35.87)*	3359.26 (41.83)*	3400.26 (44.34)*	3537.85 (46.27)	3245.11 (44.52)*	3752.89 (49.17)	3245.54 (46.25)*	3638.47 (57.04)	3612.98 (44.20)	3666.26 (129.71)

^1^kj = kilojoules(1 kcal = 4.187 kj). Covariates: Age, gender, race/ethnicity, poverty income ratio grouped into three categories as (<1.25, 1.25–3.49, and >3.49), current smoking status (yes/no), physical activity level (sedentary, moderate and vigorous), alcohol intake (g/d), energy intake for nutrient related variables.

*Statistically different from No Snacks; with the Bonferroni correction effective p ≤ 0.0042.

**Reference group is No Snacks.

Total SFA intake of the snacking patterns ranged from 9.94 g (whole fruit) to 12.45 g (milk desserts). Total intake of SFA was significantly higher in the milk desserts snacking pattern and lower in the crackers/salty snacks, soft drinks, other grains and whole fruit snacking patterns, compared to no snacks. Total intake of solid fat was similar to SFA intake. Total intake of solid fat of the snacking patterns ranged from 39.5 g (vegetables/legumes) to 50.6 g (milk desserts).

Total intake of added sugars ranged from 15.1 g (whole fruit) to 28.7 g (soft drinks). Total intake of added sugars was significantly higher in the majority of the snacking patterns, except the snacking patterns, miscellaneous snacks, alcohol, and coffee/tea, compared to no snacks. Total intake of sodium ranged from 3,245 mg (milk desserts and soft drinks) to 3,753 mg (crackers/salty snacks). Total intake of sodium was significantly lower in the cakes/cookies/pastries, sweets, vegetables/legumes, milk desserts, and soft drinks snacking patterns, compared to no snacks.

### Nutrients of public health concern and nutrients under-consumed

Table 3 shows the mean intake of nutrients of public health concern and nutrients that are of potential concern for under-consumption for sub-populations for the entire day. Total potassium intake ranged from 2,383 mg (soft drinks) to 3,047 mg (whole fruit). Total potassium intake was significantly higher in the miscellaneous snacks, vegetables/legumes, alcohol, milk desserts, soft drinks, whole fruit, and coffee/tea snacking patterns, compared to no snacks. Total calcium intake ranged from 788 mg (soft drinks) to 1,043 mg (miscellaneous snacks). Compared to no snacks, total calcium intake was higher for the miscellaneous snacks and lower for the soft drinks snacking patterns. Total intake of vitamin D ranged from 3.86 mcg (crackers/salty snacks) to 5.50 mcg (miscellaneous snacks). When vitamin D intake among the patterns was compared to no snacks, vitamin D intake was higher for the miscellaneous snacks only. Total dietary fiber intake ranged from 13.6 gm (soft drinks) to 19.5 gm (whole fruit). Total mean intake of dietary fiber was higher among the vegetables/legumes, crackers/salty snacks, other grains and whole fruit snacking patterns compared to no snacks. For those in the soft drink pattern, total dietary fiber was lower than no snacks. Total intake of vitamin A was significantly higher in the miscellaneous snacks, alcohol, and milk desserts snacking patterns, compared to no snacks. Intake of vitamin C was higher in the miscellaneous snacks and whole fruit and lower in the soft drinks snacking patterns, compared to no snacks. Total intake of folate was higher in the other grains and lower in the soft drink snacking pattern, compared to no snacks. Total intake of magnesium was higher in the vegetables/legumes, crackers/salty snacks, whole fruit, and coffee/tea but lower in the soft drink snacking pattern, compared to no snacks.

**Table 3:** **Consumption of nutrients of public health concern and nutrients under-consumed by snacking patterns**^**1 **^**in adults ≥19 years of age**

	**Snacking patterns LSM (SE)**											
	**Misc. snacks (n = 3,230)**	No snacks** (n = 2,853)	**Cakes/Cookies/Pastries (n = 2,180)**	**Sweets (n = 1,495)**	**Vegetables/Legumes (n = 1,524)**	**Alcohol (n = 1,572)**	**Milk desserts (n = 1,355)**	**Crackers/Salty snacks (n = 1,293)**	**Soft drinks (n = 1,088)**	**Other grains (n = 1,190)**	**Whole fruit (n = 913)**	**Coffee/Tea (n = 295)**
Nutrients of Public Health Concern												
Potassium (mg)	2883.71 (27.34)*	2612.52 (27.22)	2521.52 (23.69)	2633.29 (29.51)	3028.60 (37.96)*	2773.98 (34.49)*	2765.11 (39.83)*	2568.00 (34.26)	2383.33 (30.03)*	2660.57 (47.52)	3047.08 (45.95)*	2876.64 (55.98)*
Calcium (mg)	1043.12 (13.55)*	892.64 (13.40)	854.50 (13.01)	852.62 (12.22)	902.69 (20.99)	901.23 (18.02)	949.75 (16.76)	842.08 (17.94)	788.49 (21.91)*	939.26 (22.54)	934.96 (22.46)	958.84 (34.72)
Vitamin D (mcg)	5.50 (0.15)*	4.12 (0.14)	4.15 (0.15)	3.92 (0.13)	4.69 (0.20)	4.41 (0.20)	4.20 (0.15)	3.86 (0.19)	3.89 (0.22)	4.84 (0.33)	4.92 (0.32)	5.18 (0.47)
Fiber (gm)	15.56 (0.19)	14.98 (0.24)	15.02 (0.24)	15.58 (0.30)	18.80 (0.37)*	15.28 (0.31)	15.40 (0.32)	16.77 (0.33)*	13.55 (0.32)	16.49 (0.38)*	19.48 (0.41)*	16.22 (0.87)
Nutrients under-consumed												
Vitamin A (mcg)	656.07 (12.68)*	577.56 (12.86)	630.15 (19.46)	561.44 (17.36)	654.57 (35.65)	615.13 (19.82)*	681.73 (18.63)*	583.58 (18.97)	529.05 (35.54)	651.46 (60.74)	646.81 (23.89)	650.25 (32.91)
Vitamin C (mcg)	109.10 (3.01)*	84.34 (2.69)	81.62 (2.54)	81.39 (3.82)	87.50 (3.55)	81.61 (3.06)	87.58 (4.06)	79.93 (3.48)	68.26 (3.24)*	86.92 (5.35)	123.05 (5.03)*	74.40 (4.07)
Vitamin K (mcg)	95.34 (3.22)	94.33 (4.10)	92.44 (4.90)	91.61 (4.23)	117.49 (7.21)	101.43 (7.55)	84.15 (4.97)	90.63 (5.35)	80.85 (4.88)	96.54 (4.47)	120.76 (9.29)	111.13 (11.48)
Folate (mcg)	552.67 (8.58)	536.50 (9.96)	546.60 (11.73)	504.67 (11.16)	555.53 (12.32)	547.01 (13.91)	531.35 (15.69)	553.03 (11.52)	473.24 (10.45)*	617.87 (19.54)	572.67 (13.40)	547.89 (20.17
Magnesium (mg)	299.13 (2.63)	276.51 (2.80)	269.62 (2.95)	277.84 (3.46)	360.62 (7.46)*	290.43 (4.56)	282.62 (3.86)	292.21 (3.62)*	245.69 (3.32)*	287.32 (4.72)	307.46 (5.58)*	327.46 (11.89)*

^1^Covariates: Age, gender, race/ethnicity, poverty income ratio grouped into three categories as (<1.25, 1.25–3.49, and >3.49), current smoking status (yes/no) (adults only), physical activity level (sedentary, moderate and vigorous), alcohol intake (g/d), energy intake for nutrient related variables.

*Statistically different from No Snacks with the Bonferroni correction effective p ≤ 0.0042.

**Reference group is No Snacks.

### Overall diet quality

**Figure 2:**
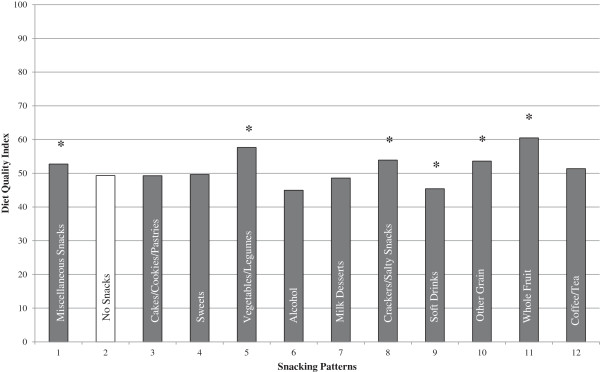
**Snacking patterns and overall diet quality (HEI-2005) for adults ≥ 19 years of age.** Covariates: Age, gender, race/ethnicity, poverty income ratio grouped into three categories as (<1.25, 1.25-3.49, and >3.49), current smoking status yes/no) (adults only), physical activity level (sedentary, moderate and vigorous), alcohol intake (g/d). Note that energy was not used as a covariate since the HEI score itself is controlled for energy. *Significantly different from no snacking (pattern 2); Bonferroni correction p ≤ 0.0042.

Figure 2 shows the Healthy Eating Index-2005 (HEI-2005) by snacking patterns. On average the HEI-2005 scores were higher for five of the snacking patterns, miscellaneous snacks, vegetables/legumes, crackers/salty snacks, other grains, and whole fruit, compared to no snacks. The HEI-2005 score for the soft drink snacking pattern was lower than no snacks. Half of the snacking patterns had a total HEI-2005 score of less than 50; which included no snacks, cakes/cookies/pastries, sweets, alcohol, milk desserts, and soft drinks.

### Snacking patterns, weight/adiposity status, and cardiovascular risk factors

Table 4 shows the relationship between the snacking patterns and weight/adiposity. Body mass index and WC were significantly lower in the alcohol and milk desserts snacking patterns respectively, compared to no snacks. For the remaining 10 snacking patterns, there was no association found with weight/adiposity. The percent of overweight/obese adults who reported >30% of energy from snacks was significantly lower compared to normal weight adults (Additional file 1: Figure S1). None of the snacking patterns showed an increased likelihood of having elevated cardiovascular risk factors when compared to no snacks (Table 5).

**Table 4:** Relationship between snacking patterns and weight by adults ≥19 years of age

	**Weight status**^**1**^	
**Snacking pattern**	**BMI LSM (SE)**	**Waist circumference, cm LSM (SE)**
Miscellaneous snacks (n = 3230)	28.39 (0.19)	97.10 (0.47)
No snacks (n = 2853)	28.54 (0.18)	97.66 (0.48)
Cakes/Cookies/Pastries (n = 2180)	28.23 (0.16)	97.66 (0.48)
Sweets (n = 1495)	28.20 (0.23)	96.80 (0.43)
Vegetables/Legumes (n = 1524)	28.34 (0.26)	96.92 (0.60)
Alcohol (n = 1572)	27.74 (0.20)^§^	97.12 (0.63)
Milk desserts (n = 1355)	28.30 (0.24)	95.72 (0.49)^§^
Crackers/Salty snacks (n = 1293)	28.49 (0.21)	97.91 (0.49)
Soft drinks (n = 1088)	28.45 (0.25)	97.78 (0.65)
Other grains (n = 1190)	28.24 (0.29)	97.20 (0.63)
Whole fruit (n = 913)	28.38 (0.38)	96.89 (0.77)
Coffee/Tea (n = 295)	29.18 (0.68)	98.24 (1.26)

^1^Covariates included age, gender, race/ethnicity, poverty income ratio, current smoking status, alcohol intake, and physical activity.

^§^Statistically different from No Snacks with the Bonferroni correction effective p ≤ 0.0042.

**Table 5:** Likelihood of having cardiovascular risk factors

	Snacking patterns*										
**CVRF**^**+ **^odds ratio (LCL, UCL)**	**Misc. snacks (n = 3,230)**	**Cakes/Cookies/Pastries (n = 2,180)**	**Sweets (n = 1,495)**	**Vegetables/Legumes (n = 1,524)**	**Alcohol (n = 1,572)**	**Milk desserts (n = 1,355)**	**Crackers/Salty snacks (n = 1,293)**	**Soft drinks (n = 1,088)**	**Other grains (n = 1,190)**	**Whole fruit (n = 913)**	**Coffee/Tea (n = 295)**
Elevated BP	1.04 (0.82, 1.31)	0.89 (0.68, 1.16)	1.02 (0.76, 1.37)	1.09 (0.77, 1.53)	0.85 (0.59, 1.23)	1.04 (0.78, 1.39)	1.13 (0.79, 1.61)	1.09 (0.75, 1.60)	0.98 (0.67, 1.43)	0.94 (0.64, 1.37)	0.97 (0.58, 1.61)
Elevated Glucose	1.08 (0.75, 1.55)	1.06 (0.74, 1.52)	0.87 (0.57, 1.34)	1.34 (0.96, 1.88)	0.86 (0.56, 1.34)	1.16 (0.78, 1.71)	1.01 (0.63, 1.60)	0.89 (0.55, 1.45)	1.30 (0.81, 2.09)	1.30 (0.73, 2.32)	0.58 (0.31, 1.11)
Elevated LDL-C	0.84 (0.61, 1.16)	1.02 (0.71, 1.45)	0.99 (0.70, 1.40)	1.12 (0.80, 1.58)	0.84 (0.53, 1.33)	0.97 (0.65, 1.44)	0.87 (0.56, 1.36)	1.13 (0.70, 1.82)	0.90 (0.53, 1.52)	0.93 (0.61, 1.42)	1.04 (0.53, 2.02)
Decreased HDL-C	0.96 (0.77, 1.19)	0.94 (0.74, 1.20)	1.23 (0.95, 1.59)	0.89 (0.68, 1.17)	0.88 (0.60, 1.29)	0.97 (0.73, 1.30)	1.08 (0.85, 1.37)	1.40 (0.98, 1.99)	0.94 (0.68, 1.31)	1.08 (0.81, 1.44)	1.15 (0.63, 2.11)
Elevated TAG	1.00 (0.73, 1.37)	0.89 (0.64, 1.23)	1.10 (0.75, 1.63)	0.97 (0.67, 1.40)	0.89 (0.58, 1.36)	0.85 (0.61, 1.19)	1.09 (0.72, 1.65)	1.29 (0.85, 1.98)	1.00 (0.62, 1.60)	0.89 (0.58, 1.36)	0.94 (0.45, 1.93)
Elevated WC	0.95 (0.77, 1.17)	0.87 (0.67, 1.13)	0.93 (0.70, 1.25)	0.88 (0.61, 1.26)	0.74 (0.55, 1.00)	0.92 (0.70, 1.21)	0.99 (0.75, 1.29)	1.03 (0.75, 1.41)	1.04 (0.75, 1.45)	0.69 (0.46, 1.02)	0.92 (0.50, 1.68)
Elevated OB	0.97 (0.78, 1.22)	0.91 (0.73, 1.15)	0.95 (0.74, 1.21)	0.89 (0.67, 1.18)	0.85 (0.60, 1.19)	0.97 (0.72, 1.29)	1.04 (0.81, 1.33)	1.03 (0.76, 1.39)	0.92 (0.69, 1.22)	0.91 (0.66, 1.24)	0.85 (0.54, 1.34)
Elevated OW	0.99 (0.80, 1.23)	1.09 (0.86, 1.38)	0.96 (0.72, 1.26)	1.00 (0.78, 1.29)	0.99 (0.75, 1.30)	1.02 (0.79, 1.32)	1.00 (0.76, 1.33)	1.00 (0.77, 1.32)	0.96 (0.76, 1.23)	0.94 (0.68, 1.29)	1.29 (0.80, 2.07)
Elevated OW/OB	0.95 (0.76, 1.20)	0.99 (0.76, 1.29)	0.91 (0.68, 1.21)	0.89 (0.66, 1.19)	0.84 (0.61, 1.16)	0.99 (0.77, 1.27)	1.05 (0.78, 1.39)	1.03 (0.75, 1.41)	0.88 (0.64, 1.21)	0.84 (0.60, 1.17)	1.12 (0.64, 1.96)

*Reference group is No Snacks (n = 2853, 13%).

**Adjusted for ethnicity, gender, age, estimated energy ratio (kcal/estimated energy requirement), poverty income ratio, Body Mass Index, Physical Activity, smoking, and Alcohol. Body Mass Index (BMI) was controlled for all of the CVRF except for BMI.

**Statistically different from No Snacks, bolded results indicate significant findings using Bonferroni correction p ≤ 0.0042.

^+^Definition of elevated CVRF: Overweight/obesity was defined as a BMI ≥ 25. Having ≥3 of the following association factors: abdominal obesity, waist circumference ≥ 102 cm (males), ≥88 cm (females); elevated blood pressure, SBP ≥ 130 mmHg or DBP ≥85 mmHg or antihypertensive medication use; reduced serum HDL-C, <40 mg/dL (males), <50 mg/dL (females) or medication use for reduced HDL-C; elevated serum triacylglycerides, ≥ 150 mg/dL or medication use for elevated TAG; elevated fasting plasma or serum glucose, ≥100 mg/dL or medication use for elevated glucose.

*Abbreviations used*: *BP*, Blood pressure; *LDL-C*, Low-density lipoprotein cholesterol; *HDL-C*, High-density lipoprotein cholesterol; *TAG*, Triacylglycerides; *WC*, Waist circumference; *OB*, Obese; *OW*, Overweight; *OW/OB*, Overweight/obese; *LCL*, Lower Confidence Level; *UCL*, Upper Confidence Level.

## Discussion

Nutrition research has traditionally focused on single nutrients in relation to health. Recently however, scientists have acknowledged the complex synergistic interactions among foods in relation to health. This has led to a growing interest in looking at dietary patterns [67-73] which makes intuitive sense, given that foods are generally not eaten in isolation. Thus, eating patterns may have a greater impact on metabolic risk factors than any single food, food group, or nutrient. This study showed that, using cluster analysis, 12 specific snacking patterns, including no snacks, could be identified in a nationally representative sample of US adults. The snacking patterns varied widely by foods consumed, nutrient contribution, and overall diet quality.

In our study, total energy intake varied by snacking pattern. Most of the snacking patterns resulted in a higher total energy intake compared to no snacks. This is consistent with other studies showing that snacking was associated with increased energy intake [11]. Three snacking patterns, alcohol, whole fruit, and coffee/tea, resulted in lower total energy intake compared to no snacks. Studies have shown that alcohol [74] or coffee/tea intake [75] was associated with lower weight or less weight gain. Other studies have shown that fruit intake was inversely associated with weight in African-American females [76,77], suggesting a lower total energy intake or increased physical activity.

Total intake of SFA was lowest for the whole fruit snacking pattern and highest for the milk desserts snacking pattern. Total intake of added sugars was significantly higher in the majority of the snacking patterns, compared to no snacks. The majority of the snacking patterns exceeded the recommendation of less than 10% of total energy from SFA [47]; none of the snacking patterns showed a mean daily intake of added sugars that exceeded The Institute of Medicine’s threshold of 25% of energy [78]. The data suggest that even those adults consuming snacks with nutrient-dense foods or beverages, such as vegetables/legumes, low fat milk, and whole fruit, may need to improve aspects of their overall diet.

Snacking has been shown to contribute significantly to nutrient intake [11,16,17], better diet quality [18], and to an increased likelihood of meeting selected food recommendations [11,16]. As shown in this study, total intake of nutrients of public health concern and nutrients under-consumed, as defined by the 2010 Dietary Guidelines for Americans [47], were higher in several of the snacking patterns when compared to no snacks.

Contrary to expectations, snacking was associated with a slightly more nutrient-dense diet. In our study, five snacking patterns had significantly higher HEI-2005 scores (when compared to no snacks). This could be a reflection that some of the foods in those snacking patterns were nutrient-dense or that poor snacking choices were compensated with healthier food/beverage choices made at the other meals. Despite the higher HEI-2005 scores found with some of the snacking patterns, the mean HEI-2005 scores for all of the snacking patterns (including no snacks) were low, suggesting that overall diet quality in adults was poor and needs improvement. These data also suggest that simply consuming a snack may not be associated with a higher diet quality, but the specific foods consumed as a snack or at meals are equally important.

Consumers may need additional educational tools on how to incorporate healthier food and beverage choices into their routine snacking behaviors. One study found that women needed information concerning snacks high in fiber and low in trans-fat [79]. Given that mothers are the primary food shoppers in the family, their lack of knowledge in selecting healthier snacks may translate into less healthier snacks being available and accessible in the homes for their children. Family-based interventions are needed for enhancing self-confidence for healthful snack selection and for overcoming barriers [79] among mothers and their children.

An important finding from this study was the lack of association between majority of the snacking patterns and weight. This is consistent with other studies [11,24-28]; however, there are studies that have shown an inverse [19,24] or positive [23] association between snacking and weight. There are several possible explanations for the lack of association between snacking patterns and weight, despite the increased energy intake associated with the snacking patterns. Snacking has been shown to promote satiety and reduce risks for obesity with improved diet quality [11,14,33] and increased intakes of fruit, whole grains, and fiber [11,14]. Snacking has also been associated with increased vigorous physical activity [16,34]; thus, the increased energy intake associated with snacking may have been balanced with increased energy expenditure during physical activity. More studies are needed to examine whether the increased energy intake reported for those who snack is an artifact of underreporting among those who do not snack or is due to increased compensation for physical activity or a lack of compensation at subsequent meals.

To our knowledge, this is the first study to show no association between snacking and CVRF. None of the snacking patterns were associated with CVRF. Possible explanations for the lack of association between snacking patterns and CVRF are less clear. A majority of the snacking patterns exceeded the recommendation of less than 10% of total energy from SFA [47]. Dietary SFA has been shown to increase LDL-C [80], and therefore has been associated with increased risk of CVD; however, recent findings question the role of SFA in CHD risk [81]. Another possible explanation for finding no association between snacking patterns and CVRF may reflect other protective nutrients (namely calcium, potassium, fiber, folate, and magnesium) that were consumed in higher amounts in those adults who snacked compared to those who did not snack. More studies are needed to better understand the complex synergistic interactions among foods and snacking patterns in relation to health.

### Limitations

NHANES is a cross-sectional study; thus, cause and effect associations cannot be inferred. Twenty-four hour dietary recalls have several inherent limitations, including that they may not reflect usual intake and are memory dependent, which may lead to under- or over-reporting; however, a single 24-hour recall is sufficient to report mean group intake [82]. Energy-dense, nutrient-poor foods and beverages, particularly when consumed as snacks, tend to be under-reported [26,83,84]. Data from this study suggested that the percent of overweight/obese adults who reported >30% of energy from snacks was significantly lower compared to normal weight adults. Whether this reflects under-reporting is debatable and needs further exploration.

## Conclusions

Twelve snacking patterns (including no snacks) were identified in a nationally representative population of adults 19+ years of age. The patterns varied in food and beverage selections and their contribution to daily intake of nutrients and diet quality. More studies are needed to confirm these findings to better understand how specific snacking patterns fit within an overall healthier eating lifestyle. Some snacking patterns may also be inversely associated with weight and abdominal obesity. Because of inconsistent evidence in the literature, there are several noteworthy findings from this study that should generate future hypotheses for further testing. Moreover, longitudinal studies are needed to further evaluate whether snacking prevents weight gain in adults.

## Abbreviations

BMI: Body mass index; BP: Blood pressure; CVRF: Cardiovascular risk factors; g: grams; HEI: Healthy eating index; HDL-C: High-density lipoprotein cholesterol; kcal: kilocalories; LDL-C: Low-density lipoprotein cholesterol; mcg: micrograms; MEC: Mobile Examination Center; mg: milligrams; MPED: MyPyramid Equivalents Database; NHANES: National Health and Nutrition Examination Survey; OB: obese; OW: Overweight; OW/OB: Overweight/obese; SFA: Saturated Fatty Acids; TAG: Triacylglycerides; USDA: United States Department of Agriculture.

## Competing interests

None of the authors have any financial or competing interests to declare.

## Authors’ contributions

Author contributions include the following: TN was responsible for conception and design, analysis and interpretation of data, and drafting the article. TN, CON & VLF were responsible critical revision of paper for important intellectual content. All authors had full access to all of the data (including statistical reports and tables) in the study, can take responsibility for the integrity of the data and the accuracy of the data analysis, and approved the final version to be published.

## Pre-publication history

The pre-publication history for this paper can be accessed here:

http://www.biomedcentral.com/1471-2458/14/388/prepub

## Supplementary Material

Additional file 1: Figure S1 Distribution of Percent Energy from Snacks by Weight Status for Adults ≥19 Years of Age Participating in the 2001-2008 NHANES. *p <0.001. Click here for file
