# Supplementary figures and images for: Snacking patterns, diet quality, and cardiovascular risk factors in adults
Source: BMC Public Health. 2014 Apr 23;14:388. doi: 10.1186/1471-2458-14-388 (PMC4108013; doi:10.1186/1471-2458-14-388)

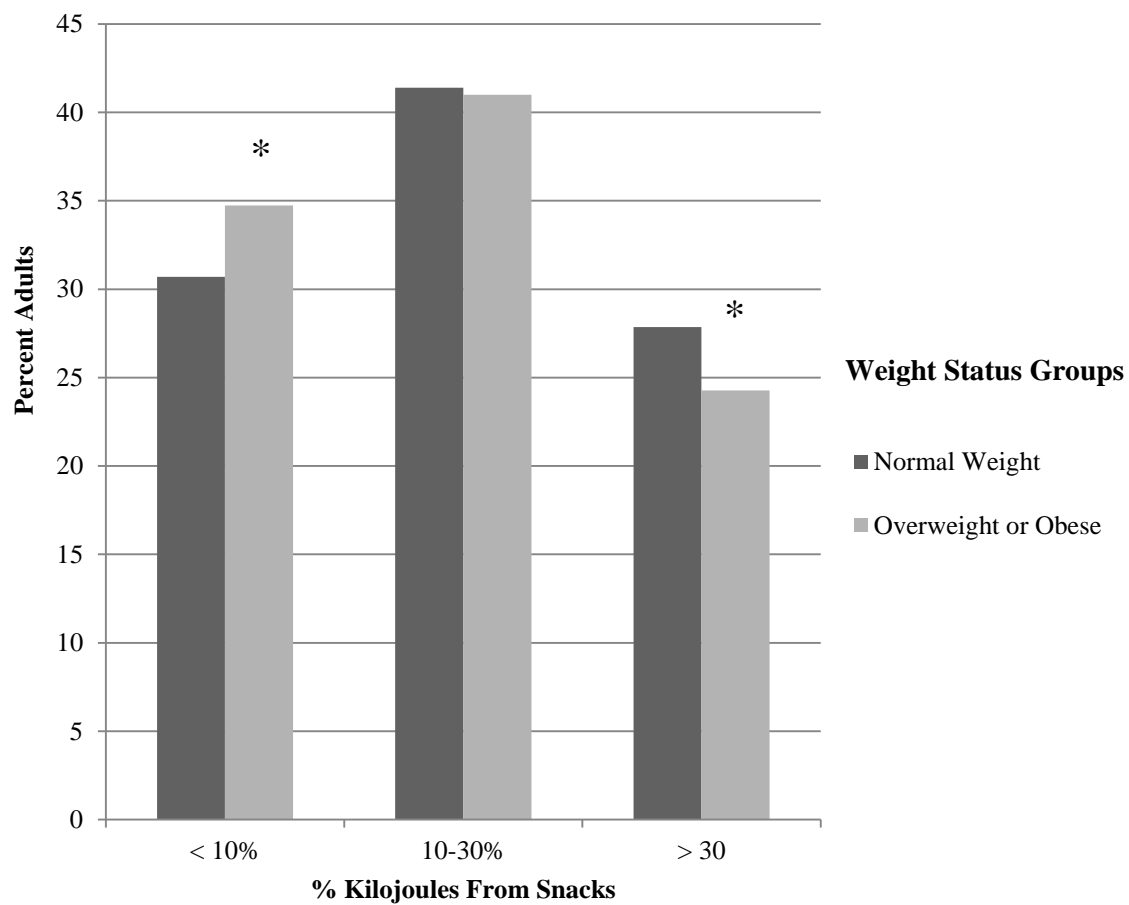

Supplement: Additional file 1: Figure S1 — Distribution of Percent Energy from Snacks by Weight Status for Adults ≥19 Years of Age Participating in the 2001-2008 NHANES. *p <0.001. [file 1471-2458-14-388-S1.pdf]
